# Towards Improved Organizational Governance of Neurotrauma Surveillance: Comment on "Neurotrauma Surveillance in National Registries of Low- and Middle-Income Countries: A Scoping Review and Comparative Analysis of Data Dictionaries"

**DOI:** 10.34172/ijhpm.2022.7554

**Published:** 2023-01-24

**Authors:** Hanna E. Schenck, Halinder S. Mangat

**Affiliations:** ^1^School of Mental Health and Neuroscience, Maastricht University, Maastricht, The Netherlands.; ^2^Department of Neurosurgery, Maastricht University Medical Center, Maastricht, The Netherlands.; ^3^Department of Neurology and Neurocritical Care, Kansas University Medical Center, Kansas City, KS, USA.

**Keywords:** Surveillance, Neurotrauma, Health Policy, Governance, Data Elements, Low- and Middle-Income Countries

## Abstract

Neurotrauma surveillance data on burden and severity of disease serves as a tool to define legislations, guide high-yield risk-specific prevention, and evaluate and monitor management strategies for adequate resource allocation. In this scoping review, Barthélemy and colleagues demonstrate the gap in neurotrauma surveillance in low- and middle-income countries (LMICs) and suggest strategies for governance in neurotrauma surveillance. We underline state accountability as well as the need for the close integration of academic and tertiary care clinical practitioners and policy-makers in addressing the public health crisis caused by neurotrauma. Additionally, multiple sources for surveillance must be included, especially in communities where victims may remain without access to formal healthcare. Finally, we offer insights into possible ways of increasing the visibility of neurotrauma on political agendas.

## Introduction

 Barthélemy and colleagues present an overview of existing national neurotrauma registries in low- and middle-income countries (LMICs) in an attempt to outline current practices for collection of neurotrauma surveillance data.^1^ Their work identified a total of 16 national registries in LMICs through a systematic review of the literature, cold contacts of ministries of health, and of global health actors involved in this field, and demonstrates disparities in neurotrauma surveillance systems in LMICs. Whilst neurotrauma registries for the vast majorities of LMICs could not be identified, amongst those that were accessible, there were absences of ‘Minimal Dataset for Injuries’ as proposed by the World Health Organization (WHO). However, as the authors themselves suggest, absence of scientific publications from registries may not necessarily imply absence of registries, as publication volume, especially in neurotrauma from LMICs is known to be low.^2^

 The presence or absence of national neurotrauma registries may reflect whether neurotrauma is a top national public health concern, whether addressing neurotrauma surveillance is feasible and constrained, or whether actors who should be pushing this into national health plan are not empowered. Absence of such surveillance data then impacts legislation and policy formulation on disease prevention. As stated by the authors, neurotrauma is more than about accidents, but massive economic costs, lost disability-adjusted life years, and loss of young citizens of the future generations.

 Here we discuss disease surveillance in relation to its purpose and its place within health systems. In light of this, we will provide arguments that underline the “political” nature of neurotrauma surveillance and the necessity of national governance in neurotrauma surveillance. Notwithstanding the heterogeneity of political landscapes in LMICs, we argue that instigation from (inter)national civil society organizations like the WHO, World Federation of Neurosurgery (WFNS) as well as continental, regional and national medical associations can help leverage initiatives to improve neurotrauma care and assist governments in implementing measures to optimize neurotrauma surveillance. Finally, we reflect on the proposed recommendations from the article of Barthélemy et al and discuss how addressing the tremendous gap in neurotrauma surveillance will require a combination of bottom-up and top-down measures.

## Surveillance: Protection, Monitoring and Accountability

 The authors refer to neurotrauma surveillance as “an ongoing systematic collection, analysis and interpretation of data on neurotrauma, and a close integration of that data with its timely dissemination to government offices and health ministries that are accountable for injury control and prevention.” The French word “surveiller,” observing and keeping an eye on, relates to one of the primary goals of surveillance within a nation state, which is that of watching for any threat to the welfare of its citizens or their health. ‘Health surveillance’ is a well-known term in the arena of public health, where it serves ministries of health and nation-states to timely identify and react to acute outbreaks of communicable diseases, or the slow trend of increasing non-communicable diseases such as hypertension, diabetes, and obesity. Surveillance systems have been particularly effective in preventing and managing infectious and tropical diseases in LMICs and have been widely supported by international organizations like the Task Force for Global Health and regulated by the International Health Regulations from the WHO. Surveillance in neurotrauma is less well known and not nearly high enough on the agenda of various stakeholders due to pre-occupation with communicable diseases in LMICs. The establishment of disease specific registries is used not only to track incident diseases but also as an evaluation and monitoring tool for existing policies for both policy makers and healthcare workers, and provides insights into success and failures of different management strategies, therefore serving as a closed-loop feedback mechanism. Finally, and as mentioned in this article, surveillance through registries allows collection of local data that can be used to develop interventions tailored to specific environments which may be different to interventions that may be used in other environments such as in high-income countries (HICs). We will discuss the two first aspects of surveillance in neurotrauma and elaborate on the importance of governmental involvement in addressing the burden of neurotrauma.

## State Accountability in Neurotrauma Surveillance

 Neurotrauma in LMICs most-commonly results from road traffic accidents and violence, which are modifiable and societal factors. The incidence of traumatic brain injury from road-traffic accidents is increasing in LMICs related to the increase in motor vehicular traffic but also lagging road safety, road lighting, passenger restraint and protective systems, and establishment and enforcement of corresponding laws for driving under the influences of alcohol and drugs. Only 28 countries worldwide (7% of the world population), have adequate laws regulating speeding, drink-driving, helmet and seat-belt-use.^3^ Likewise, neurotrauma from violence exceeds that in HICs, be it regional-conflict related, or day-to-day inter-person violence. Young males are invariably the victims, and suffer loss of ability to earn a livelihood, contribute to society, as well as likely deplete existing financial safety nets for treatment. Thus, neurotrauma for all purposes is a public health crisis, and should be tackled by health authorities; though for this to happen in an organized programmatic way, surveillance data is necessary. Surveillance of neurotrauma must be recognized as a tool to protect citizens from preventable injuries and must rise from the societal and economic incentive of keeping citizens healthy. In that respect, it is the role of a nation-state to keep track of how, where and when these neurotraumas occur to inform policies for prevention and resource allocation for management of these traumas. Examples of national registries in the article from Barthélemy et al illustrate how governments have sought to understand the causes of trauma by tracking injuries and have sought to implement more legislations and risk-specific interventions.

 Underlining state accountability in neurotrauma surveillance should also remind neurosurgeons and healthcare workers of the importance of advocating for change and of vocalizing their needs in political settings. The growth of international civil society organization like the WFNS, the European Association of Neurosurgeons, the American Association of Neurological Surgeons, the Continental Association of African Neurosurgical Societies, Latin American Brain Injury Consortium provides neurosurgeons with a stage on which they can advocate for change and propose solutions. These transnational organizations have the manpower, tools and skills to produce data and to reveal what governments may know yet ignore. An example illustrating this is how the WFNS developed an interactive map illustrating the neurosurgeon density per country, demonstrating the great disparities in neurosurgical healthcare and serving as a tool to confront ministries of health with the hard facts.^4^ The Lancet Neurology Commission on Traumatic Brain Injury makes recommendations on establishing trauma registries to map epidemiology of TBI and fund research in LMICs.^5-7^ As such, professional academic organizations have the power to shape and democratize political agendas thereby catalyzing governmental action (see Figure illustrating the interactions of healthcare workers with different bodies that can lead to improvements in neurotrauma care).

**Figure:**
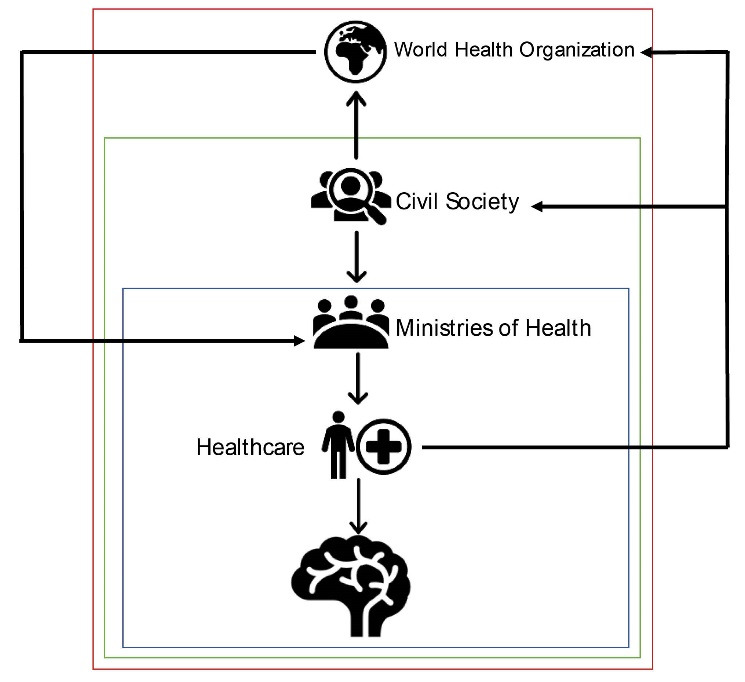
Schematic Representation of the Different Possible Interactions Between Healthcare Workers and Institutions Enhancing Neurotrauma Care Through Bottom-up and Top-Down Advocacy.

## Efficiency in Surveillance Systems

 From the bottom-up, the most-important element is collecting and entering data into a surveillance system. One of the recurring barriers to successful data entry is appointing staff responsible for data collection and ensuring continuity in collection of data. Motivation, lack of insight into the purposes of data collection, inadequate resources, multiple differing tasks, clunky information technology systems, can be real barriers to implementing neurotrauma registries due to inefficient organizational governance. Often, data collection is dependent on individuals employed for this task rather than organized regional and national integrated teams and systems. Thus, the system is not resilient to the absence of individuals which can have a domino effect.

 The task of data collection in itself is a limitless one as to where does one collect all this data from. While the registries described are those based on hospital admissions, they yet inconveniently exclude patients that do not have any interaction with the medical system. Not long ago, concussions were not seen as routinely as they are now yet constituting 90% of TBI burden in HICs due to increased awareness. Other sources of neurotrauma to be included would include police sources, community leaders, performing verbal autopsies in unknown causes of death in communities, pathological reports, death records, and funeral agencies, in addition to hospital records. While it may appear daunting, data for several illnesses, pregnancies, infant immunizations and deaths, are already collected in such manner within communities by the community health workers in many LMICs and may be a source of partnership.

 Utilizing the ‘Minimal Dataset for Injuries’ WHO framework may be an initial starting point. Leveraging technology and utilizing handheld devices (given the vast population in LMICs has access to cellular devices and network), may be another step to streamline data collection in many domains. Data may be transmitted in an encrypted form to central governmental data servers and circumvent paper forms which either may be misplaced or at the least require someone to re-enter the data into an electronic form. Minimizing redundant work is key to making this process less arduous. Additionally, every primary healthcare clinic, district, and tertiary hospital could contribute data into a centralized system. As an example, a university-based research team collected data on 1635 TBI patients undergoing surgical interventions across 150 hospitals in 57 countries over 14 months as part of a research study, by utilizing such approaches.^8^

 It is feasible, even with a small team to organize meaningful surveillance system. The key element is to raise awareness about the benefits of implementing surveillance systems for healthcare workers and governments alike. Global health organizations can play a key role in advocating the known benefits of surveillance systems and in supporting health institutions in the process of registry implementation. It should become clear to ministries of health that any investment made in the healthcare sector is a lost investment if not evaluated and monitored.

## The Need for Effective Health Advocacy by Healthcare Providers

 Barthélemy et al present in their work a few recommendations for the implementation of registries such as using readily available data dictionaries from the WHO or have the modality of data collection be context-specific and dependent on the available resources. Moreover, the authors state that neurosurgeons have a key role in informing the process of data standardization and improvement. A structured and explorative review from Bommakanti et al explored the barriers to trauma registry implementation in LMICs and revealed limited resources, unfavorable health policies and a lack of trauma education as being the main obstacles to registry implementation.^9^ Implementing a neurotrauma surveillance system requires a minimum standard of infrastructure that is lacking in the vast majority of hospitals caring for victims of neurotrauma. Healthcare workers in resource-constrained environments face shortages in human resources and often high volumes of patients, a setting in which concerns for data collection are subjacent to all of their other priorities. Moreover, data collection requires a minimum level of understanding of neurotrauma scoring systems for correct analysis and interpretation of patient data, which may not be met.

 As with educational systems elsewhere, physicians and surgeons alike, are not taught health advocacy, a gap in the health services educational curriculum. Not only should the neurosurgeons be instrumental in devising contents of neurotrauma surveillance systems, but they must also bridge the medicine-politics gap, advocate for key reforms and educate politicians on key healthcare related priorities. For if not them, then who?

 These actors can combine the knowledge of effective management of neurotrauma with the moral authority arising from experiencing first-hand the tragedies of inadequate care for neurotrauma patients. In recent years, neurosurgeons from all around the world have increasingly attended the World Health Assembly, a decision-making body of the WHO attended by its 194 member states, and allowed for the emergence of new resolutions that provide opportunities for improved access to neurosurgical care.^10^ In 2019, a new resolution on emergency care systems ensuring timely care of the ill and injured was passed, further increasing the mandate for LMICs to prioritize emergency medicine in trauma.^11^ The World Health Assembly 73 passed several resolutions on how to frame efforts to strengthen surgical systems^12^ and the Lancet Commission on Global Surgery has outlined a policy brief on “Action and Opportunities for Low-Income and Middle-Income Countries” on evidence and solutions for achieving health.^13^ Recently, a body of international neurosurgeons from HIC and LMICs have developed comprehensive policy recommendations for head and spine injury in LMICs, designed to assist policymakers in integrating neurotrauma care at a national level.^14^ The drafting of the Bogota Declaration for Global Neurosurgery is yet another example of the power of neurosurgeons to promote advocacy for improvement in access to neurosurgical care. All the above prove the strength of healthcare workers in defining a political agenda, and in facilitating the process of integration of neurotrauma care on the political agenda of nations that may be fragmented, especially in LMICs (Figure).

 It may indeed be that in LMICs, development of public health is the most important area of research to be able to successfully demonstrate the benefits of prevention of neurotrauma and of organized emergency systems for the management of neurotrauma.^15^ With adequate governance, personnel may be trained for specific purposes of disease surveillance, systematically reporting and collecting data. These trainings would be shorter and therefore less costly to governments, and could alleviate the pressure on current health professionals to fulfill the task of data collection, therefore ensuring more efficient and reliable data collection as well as healthcare provision. Finally, we want to underline the impact of initiatives in the field of global neurosurgery like capacity building through twinning.^16^ Twinning of healthcare specialists from HICs and LMICs represent a viable and effective way for the implementation of neurotrauma registries but may distract from long-term priorities and undermine formation of robust institutions. Therefore, such partnerships must constantly re-evaluate their role in not just providing infra-structure but going one-step further in making the process self-reliant.

## Conclusion

 To summarize, we believe it is in the remit of governments, to protect their citizens’ health by ensuring surveillance of diseases as a national priority. Amongst non-communicable diseases, neurotrauma has gained significant priority by virtue of the cost it exacts from society and the young population it affects disproportionately. It is from these registries, that societal and modifiable risk factors of neurotrauma will be determined and modified. This top-down approach should be a response to a bottom-up advocacy from health-care workers empowered to operate as actors in public health and represented in professional academic organizations.

## Ethical issues

 Not applicable.

## Competing interests

 Authors declare that they have no competing interests.

## Authors’ contributions

 HS and HM both equally contributed to the concept and drafting of the manuscript.
